# Quantification of image texture in X‐ray phase‐contrast‐enhanced projection images of in vivo mouse lungs observed at varied inflation pressures

**DOI:** 10.14814/phy2.14208

**Published:** 2019-08-23

**Authors:** Frank J. Brooks, Sean P. Gunsten, Sunil K. Vasireddi, Steven L. Brody, Mark A. Anastasio

**Affiliations:** ^1^ Department of Bioengineering University of Illinois at Urbana‐Champaign Urbana Illinois; ^2^ Department of Internal Medicine Washington University School of Medicine St. Louis Missouri; ^3^ Heart and Vascular Center MetroHealth Campus at Case Western Reserve University Cleveland Ohio

**Keywords:** image texture, lung imaging, statistical learning, X‐ray phase‐contrast imaging

## Abstract

To date, there are very limited noninvasive, regional assays of in vivo lung microstructure near the alveolar level. It has been suggested that x‐ray phase‐contrast enhanced imaging reveals information about the air volume of the lung; however, the image texture information in these images remains underutilized. Projection images of in vivo mouse lungs were acquired via a tabletop, propagation‐based, X‐ray phase‐contrast imaging system. Anesthetized mice were mechanically ventilated in an upright position. Consistent with previously published studies, a distinct image texture was observed uniquely within lung regions. Lung regions were automatically identified using supervised machine learning applied to summary measures of the image texture data. It was found that an unsupervised clustering within predefined lung regions colocates with expected differences in anatomy along the cranial–caudal axis in upright mice. It was also found that specifically selected inflation pressures—here, a purposeful surrogate of distinct states of mechanical expansion—can be predicted from the lung image texture alone, that the prediction model itself varies from apex to base and that prediction is accurate regardless of overlap with nonpulmonary structures such as the ribs, mediastinum, and heart. Cross‐validation analysis indicated low inter‐animal variation in the image texture classifications. Together, these results suggest that the image texture observed in a single X‐ray phase‐contrast‐enhanced projection image could be used across a range of pressure states to study regional variations in regional lung function.

## Introduction

Small rodent models have become crucial to the research of lung physiology and pathology (Kiessling et al., 2017; Pinar and Jones, 2018). Owing to the importance of the pathologic changes in airflow and lung volumes in disease, there is an interest in assessing these functions in experimental models. Because spirometry and tidal volume techniques provide only summary measurements of the entire lungs, there is continued interest in image‐based techniques for assessing regional lung physiology (Pinar and Jones, 2018) via assessing the physical properties of the lung microstructure under various imposed conditions. The current reference standard for small animal lung imaging remains computed tomography (micro‐CT) (Clark and Badea, 2014; Badea, 2018). Although this imaging modality has provided tremendous insight, there are significant limitations. A typical micro‐CT scan requires the acquisition of hundreds of individual tomographic views. This increases the total radiation dose delivered to the subject and thus can preclude some longitudinal experiment designs. Another key disadvantage of micro‐CT (and radiography) is that the relatively low image contrast for soft tissue within and near the lungs complicates the study of lung microstructure. This limitation might be overcome by the development of x‐ray phase‐contrast enhanced (XPCE) imaging specifically for the lungs (Bravin et al., 2013; Dubsky and Fouras, 2015).

The contrast in a conventional medical X‐ray image results from differences in energy absorption within the object imaged (see Refs. [Endrizzi, (2018); Gureyev et al., 2009; Pelliccia et al., 2018] for in‐depth reviews of X‐ray phase‐contrast imaging). Thus, the interpretation is that X‐rays traveling in straight lines are attenuated to varying degrees as they pass through different parts of the object before reaching the detector plane. However, under specific conditions on the coherence of the incident X‐ray beam, the X‐rays are more wavelike and therefore can refract within the object. Depending upon the object properties, such refractions can be detected as a change in the phase of the X‐ray waves. Collectively, the phase changes can yield a distinct modulation of intensity at the detector plane but the means of isolating those phase effects depends upon the specific imaging scenario. In a propagation‐based XPCE imaging system, the additional phase contrast is largest at the edges of the object. Here, an edge is any interface between materials of differing refractive decrement (which is the real part of the complex refractive index). Such interfaces are present not only at the geometric boundary defining the lung, but at every alveolus, acinus, and larger airway throughout the entire organ. Thus, one expects to see more well‐defined lung structure in a high‐magnification, propagation‐based XPCE image than in a traditional radiograph or single tomographic view.

There have been several recently published studies of XPCE imaging of small animal lungs (Garson et al., 2013; Leong et al., 2013; Kitchen et al., 2015; Larsson et al., 2016; Gradl et al., 2017; Lovric et al., 2017; Preissner et al., 2018). Some have shown striking images of alveolar‐level structure of both intact and sectioned mouse lungs obtained postmortem or ex vivo. Recently, dynamic XPCE‐CT images of in vivo mouse lungs have been published (Preissner et al., 2018). Across significant variations in animals, protocols, and imaging system parameters, many of the published XPCE lung images exhibit a self‐evident texture. In general, “image texture” is a subjective notion of a visibly perceptible grain, pattern or sense of nonrandom arrangement conveyed by locally correlated intensity variations within a larger image. Although there is almost always some sort of texture perceptible around every point throughout an entire XPCE image of a thorax, the texture within an air‐filled lung is distinct from that of regions far outside of the lung. To the best of our knowledge, the only published analyses of XPCE lung image texture are based upon statistics derived from the Fourier power spectrum of the images (Leong et al., 2013; Leong et al., 2014; Kitchen et al., 2015). The relationship between the power spectrum and the size of spherical phase‐altering objects has been modeled (Leong et al., 2013), but only for very specific assumptions about the X‐ray beam, the detector, the imaging geometry, and the material properties of the objects. The assumptions and mathematical analyses themselves pose challenges to widespread preclinical use of the image texture information clearly visible in XPCE lung images. Furthermore, we have found no published works describing how the distinctive image texture in XPCE lung images varies regionally.

We hypothesize that quantifiable differences in single, XPCE‐projection images of small animal can be detected repeatably via a straightforward image texture analysis. We acquired XPCE images of live, anesthetized mice in an upright position during mechanical ventilation at various pressures. We analyzed the observed image texture generically, without presumption about the physical cause of that texture or how it is sampled. Our results indicate that the image texture observed in a single XPCE projection image of mouse lungs varies regionally, is distinct from expected variations in X‐ray attenuation, and can indicate the expansion state of the lungs in vivo despite the presence of strongly attenuating objects such as the heart, mediastinum, and ribs.

## Methods

### Animal preparation

The Washington University Institutional Animal Care and Use Committee approved all animal studies. Male and female C57BL/6J mice (Jackson Laboratory), 8‐12 weeks old, were imaged. Mice were sedated with an intraperitoneal injection of 2,2,2‐tribromoethanol in 2‐methyl 2‐butanol (Avertin, Sigma), 250 mg/kg, and the hair covering the thorax was removed using containing depilatory lotion (Nair, Church & Dwight) containing calcium and sodium hydroxide. This was done in an effort to reduce potentially confounding texture in our projection images. A 20‐gauge, 1 inch plastic catheter was placed into the trachea by the oral route, aided by a fiber‐optic illuminator (BioLite, Braintree Scientific). The catheter was equipped with a cone‐shaped gasket to maintain proper position in the trachea and to enhance the air seal within the trachea and posterior pharynx (Safety Wedge, Kent Scientific). The catheter was attached to tubing connected to a ventilator (SAR‐1000, CWE, Inc) and ventilation supported at a rate of 120 breaths/min using a tidal volume of 15 mL/kg and 100% oxygen. Sedation was maintained with continuous administration of 1‐3% isoflurane at a flow rate of 1‐2 L/min. The mouse was held in the vertical position by sutures around the incisors to maintain the head in the upright position and each limb held with paper tape to a custom‐made acrylic frame with an opening to allow free movement of the thorax and abdomen during respiration. After imaging, the mouse was determined to still be deeply sedated by lack of reaction to a toe pinch and then was sacrificed by cervical dislocation. Lungs were then removed, fixed, and sectioned into slides such that the morphology could be analyzed for any abnormalities due to the imaging procedure. All lungs appeared to be normal (data not shown).

### Bench‐top X‐ray phase‐contrast image acquisition

**Figure 1:**
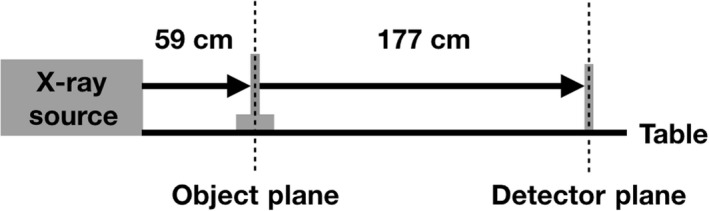
Schematic of our propagation‐based XPCE imaging system. The extended propagation distance from the object to the detector yields a higher magnification factor (here, 4×) over traditional radiography or CT imaging and permits resolution of the characteristic edge‐enhancement associated with X‐ray phase‐contrast imaging

All imaging experiments were conducted in our laboratory hosted at the Mallinckrodt Institute of Radiology at the Washington University School of Medicine. The exit window of X‐ray source is mounted at one end of an approximately 2 meter optical table (schematic, Fig. 1). The X‐ray beam was produced by a liquid‐metal‐jet‐anode, micro‐focus source using an Indium‐based alloy as the anode material (MetalJet D2+, Excillum AB). The source was operated at 100 W intensity at 70kVp and with a Beryllium exit window. For these settings, the effective X‐ray spot size is approximately 10 μm in diameter. The exposure time for a single projection image was 4 seconds. The source‐to‐object distance was 59 cm and the object‐to‐detector distance was 177 cm. In this geometry, the entire whole lungs usually are visible within the field of view (FOV) of our 4096 × 4096 pixel CCD camera (QuadRO, Princeton Instruments). Actual images output are 1998x2048 pixels at 30 micron pixel pitch with 16‐bit color depth. In the case of imaging an uncommonly large mouse, a narrow, horizontal band (∼1 mm) within the base of the lung was excluded from the FOV. The approximate total dose delivered to the mouse is 8.4mGy per projection image as measured via an Unfors NED‐30 dosimeter (Unfors Instruments AB).

During each 4‐sec exposure, the breath was held constant via the mechanical ventilator at one pressure. After approximately 30 sec of respiration while ventilated, a new pressure was selected and the mouse imaged again. Projection imaging was repeated such that precisely three images at each pressure—6, 8, 10, or 12 cm H_2_O—were acquired in random order. During the total acquisition time, the pressure at the mechanical ventilator was visually monitored and no substantial drops in pressure were observed.

### *Image correction*, *enhancement*, *and labeling*

All image processing described in this subsection was done using Fiji v1.0 (Schindelin et al., 2012) unless noted otherwise. An image acquired with our system is saved as a 16‐bit grayscale uncompressed TIFF image to which a standard flat‐field correction is applied. For convenient processing, all images were cropped to 1984 × 1984 pixels such that the cropping region was visually centered horizontally and always included the apex of the right lung. Due to our particular detector setup, the images were rotated 180° such that the lungs appear upright as the X‐ray beam travels from anterior to posterior. Because the image contrast varied considerably across mice, these image data were standardized as follows. One key image of subjectively good quality was selected. To remove outlier intensity values, the image was lower thresholded at the 0.01th percentile of all grayscale values and similarly upper thresholded at the 99.9th percentile. The grayscale intensity histogram of this contrast‐enhanced image was used as the template for all other images in a histogram‐matching scheme similar to that described in Ref. Sonka et al., (2015) that was implemented via a Python script (www.python.org). The result is that all the images have identical grayscale intensity distributions. Using the polygon selection tool, the lowest pressure image for each mouse was segmented manually into five distinct regions‐of‐interest (ROIs): the rib cage, the right lung, the left lung, the heart, and the diaphragm. All such defined ROI boundaries are outer envelopes. Thus, for examples, the rib cage includes the lungs and lungs include some ribs appearing in projection to overlay the lungs. A Fiji macro was run to spline‐fit the ROIs and perform set arithmetic to define additional regions of lung–heart overlap within the rib cage. After this, the heart region was not retained but a new region was computed to comprise all pixels outside the rib cage, that is, the complement of the entire image and all ROIs defined previously. For each mouse, minor manual adjustments were made to the ROIs to accommodate lung expansion with changing pressure. The result is a set of 32 RGB images with each ROI differently colored. Combinations of these ROIs were used as training labels in various image classifiers described below. An example for one mouse is provided (see online Supplement https://doi.org/10.7910/DVN/WULRFA) (Brooks, 2019).

### Computation of image statistics

All computation of statistics from the images was done using custom code written in Python v3.6.4 (www.python.org) using recent versions of the NumPy, SciPy, scikit‐image, and Pillow packages. The computation of all statistics described below, in total, took less than 1 min per image on a desktop computer. Images in four different feature modes described below were convolved with a 32 × 32‐pixel window in strides of 16 pixels. We chose the window size of 32 × 32 pixels because these windows are large enough such that variations in texture can be seen within them while still being small enough to fit inside the narrow rib regions. Thus, the texture that is observed to pass through the ribs is well‐sampled by many windows that clearly are: not rib, partially rib, or entirely rib. Within each window, either the median and interquartile range or the mean and standard deviation was computed, as appropriate. The result is a set of eight summary statistics for each window. These statistics, along with three others also described below, serve as the predictors in our image classification models.

The feature modes are: identity, skeleton, gradient, and Laplacian. These modes are computed as follows. Identity mode is the corrected and histogram‐matched image data as described previously but normalized to the range of the grayscale common to all the images. Skeleton mode is computed by first applying the local Otsu threshold (square width 32 pixels) to binarize the identity mode image and then thinning the resulting structure to single‐pixel width. The window mean of this mode is a representation of the sparsity of local structure. Gradient mode is the magnitude of spatial gradient of the entire identity mode image and serves as an extensive measure of window heterogeneity. Laplacian mode is the Laplacian of the entire identity mode image after single‐pixel‐radius (*σ* = 1) Gaussian blurring; even relatively weak edges are highlighted in this mode.

For the identity mode images, the Fourier power spectrum of each 32 × 32‐pixel image window was computed and multiplied by a 32 × 32 pixel, Gaussian bandpass filter (2 < *σ* < 8) to exclude the constant‐ and high‐frequency components. The sum of the logarithm of the filtered power spectrum was recorded as a measure of the total signal power and was used as a predictor.

The probability distribution function of texture orientation angles was computed for the Laplacian mode images using a directional filtering scheme similar to that described by Chaudhuri et al. (1987). The “orientation” was defined to be the mode of this distribution and the “horizontality” to be the total probability of an angle being between –45 and 45° (i.e., of being more horizontal than vertical). This value was rescaled such that values near zero indicate no strong texture orientation while values near –1 or 1 indicated strongly vertical or horizontal orientations, respectively. We note that there are other descriptions and definitions of texture orientation (Ravishankar Rao, 1990; Jähne, 2004) and that we made no distinction between angles separated by 180°. For example, textures along 90° and –90° were considered to be equivalently vertical.

### Image classification

For each image, a set of summary statistics was computed for every 32 × 32‐pixel window as described above. For our 1984 × 1984 pixel image size, and in strides of 16 pixels, there are 15129 such windows. For a given image texture classification task, a training label was assigned to each window using the ROIs described previously. The windows did not always align perfectly with the smooth boundaries of the chosen ROIs. In such cases, if a window was not at least 95% one label, that window was excluded from training. Two distinct image texture classification tasks are described below; both were done using the random forest algorithm as computed in R v3.5.1 (R Core Team, 2018) via the randomForest library v4.6‐14 (Liaw and Wiener, 2002). The number trees was set to 128 and all other relevant parameters left at the default value.

The automated image segmentation of regions likely to be lung was set up as a binary classification task. Large regions of the image visually identified to be within the bulk of a lung (left or right) were given label 1 and regions certainly not‐lung—such as those in the spine or outside the rib cage—were given the label 0. On average, there are about 3750 label 1 windows per image. Note that label 1 includes many windows that are chiefly rib because the ribs are seen in projection to overlap the lungs. Image regions that could not definitively be identified as one or the other label, such as lung regions that might partially overlap the heart or diaphragm, were excluded from training. The summary statistics for each image window were input as predictors into the random forest algorithm with the lung (1) versus not‐lung (0) labels as outcome factors. The result is a trained, numerical classifier that can be applied to the same set of image‐derived statistics but computed for other, similar images. The accuracy of the classifier was cross‐validated by first training on data from all but one “hold‐out” mouse, applying the trained classifier to the hold‐out data and then computing the percentage of correct window classifications from the previously defined ROI labels. This was repeated for each of the mice, in turn, resulting in eight distinct accuracy measurements for one ventilation pressure. Here, accuracy is the sum of the diagonal of the 2 × 2 confusion matrix divided by the matrix total. In other words, the accuracy is the ratio of the number of correct classifications (of either label) to the total number of classifications attempted.

The relation of observed image texture to inflation pressure was set up as a multiclass classification task. Here, the outcome factors are the pressures 6, 8, 10, and 12 cmH_2_O and the predictors again are the summary statistics previously described. Note that this is an independent classifier which is free to use the same data in different ways. Accuracy was defined and cross‐validated as described previously.

In every case of training, labels were balanced exactly and all validation data were excluded from training. Additionally, although the random forest algorithm commonly is employed without the scaling or centering of predictors, we believe that both are necessary for the particular cross‐validation scheme employed. Therefore, all predictors were transformed to z‐scores on a per mouse basis prior to training.

### Lung texture enhancement

For illustration purposes, a texture‐enhanced viewing mode was created using Fiji, as follows. The FeatureJ plugin (Meijering, [Link]) (https://imagescience.org/meijering/software/featurej/) was used to compute the Laplacian of the grayscale image. The result was converted to 16‐bit mode and 0.1th and 99.9th percentile thresholds applied to remove intensity outliers. The “Membrane Projections” (Arganda‐Carreras et al., 2017) filter of the LungJ plugin (Wollatz et al., 2016) was applied to the Laplacian image using a membrane size of 1 and patch size of 31 pixels; the maximum intensity projection image was selected. The image contrast was manually enhanced and a false‐color look‐up table (FireLUT) was applied.

### Atlas images

For another illustration, images of lung regions across different mice were mapped to a common lung region using the bUnwarpJ plugin (Arganda‐Carreras et al., 2006) for Fiji. Several landmarks were placed manually on the source image—for example, at the corners of the convex hull of a lung region mask—and then manually moved to the corresponding location on the target image. The resulting B‐spline transforms for each source mask were saved to files and then applied to new images via a Fiji macro. We note that mapping to an atlas image is for illustration purposes only is in no way required for any part of the method presented.

## Results

### XPCE lung image texture

**Figure 2:**
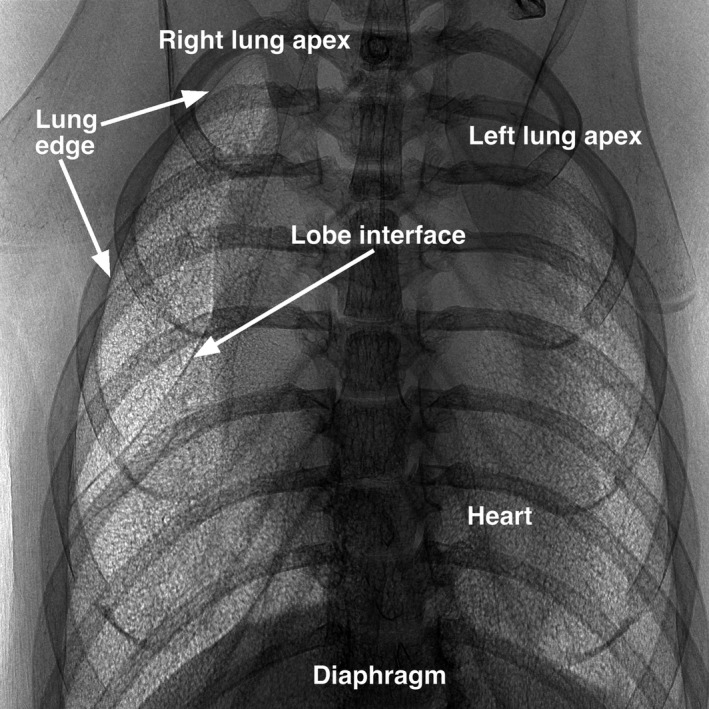
An extended‐geometry, mixed‐mode, X‐ray projection image of in vivo mouse lungs imaged with breath held at 10 cmH_2_O. The original image is 1984 × 1984 pixels and the vertical edge corresponds to approximately 60 mm in length

In vivo images of the thorax of a total of eight mice (*N* = 8) were obtained in the upright position using our XPCE system (e.g., Fig. 2). The ventilation pressure was 10 cmH_2_O and the vertical edge of the image corresponds to about 60 mm. There are several key observations to make about the projection image. Near the right apex, for example, the edge of the lung is well‐defined as a darkened, curved boundary. This boundary may be followed visually from the apex, along the lateral edge of the rib cage, toward the base of the lung. On the medial side of the apex, the lung boundary may be inferred but is less well‐defined as it overlaps (in projection) with the heart and mediastinum. Within the boundary, the image texture is manifestly distinct from that outside the boundary. In general, the interior texture is subjectively rougher than is the exterior texture. Note how the interior texture is uninterrupted by the overlaying ribs and appears to uniquely colocate only with the expected position of the lung as that is inferred from the anatomy. This can be seen again on the left side of the thorax where the rough image texture overlaps the heart which is seen as a dark, vaguely defined region at the medial side of the left lung. Recalling that the heart beats during the four‐second image acquisition, the darkened region represents the average position of the heart during breath hold.

**Figure 3:**
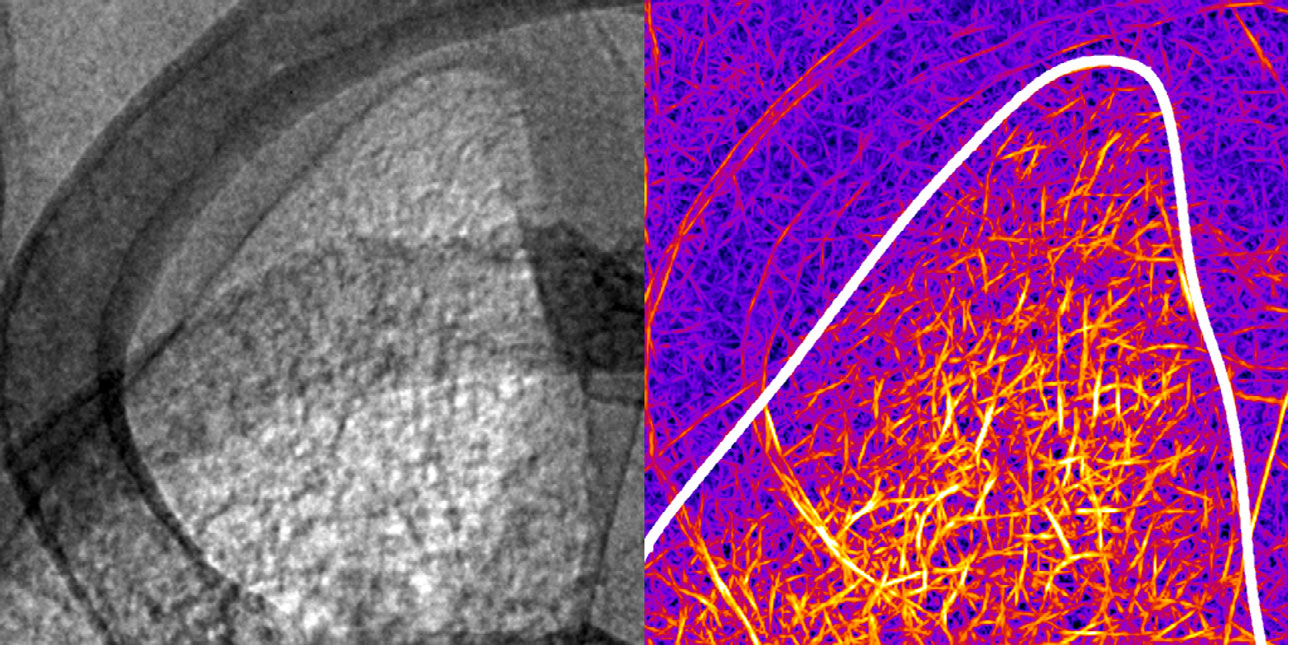
Inset from Figure 2. Shown at the left is a region near the right lung apex. In this figure, the intensity contrast is manually enhanced. At the right, the same region but with the texture contrast enhanced via a purposely applied set of filters. The images have been rescaled; the vertical edge corresponds to about 8 mm in length

The difference between the lung texture and the textures throughout the rest of the image can be seen near the right apex (Fig 3). The display window for the grayscale image on the left was manually adjusted to enhance intensity contrast. Again, the dark edge defining the apex is clearly identifiable. Interior to that boundary, the image intensity appears brighter and the image texture appears rougher. Note the relative smoothness of regions superior to the apex boundary and those outside the rib cage at top left corner of the image. A key observation is that the exterior smoothness and interior roughness each pass through the darkened ribs, thus indicating that the texture contrast is distinct from the intensity contrast. The image at the right of Figure 3 is a texture‐enhanced view of the image at the left. Note that this is an applied viewing mode where the color intensity does not necessarily correspond to any clinical quantity. In this viewing mode, it is clear that the image texture is visually distinct across the lung boundary that is indicated as a white curve. The feature modes described in the Methods section each at least partially capture different aspects of the perceived image texture. For example, the statistics computed for the Laplacian mode summarizes the total intensity and intensity variance of persistent edges of the sort that are manually enhanced in Figure 3 (right).

### Image segmentation

Automatic delineation of the image into regions of lung or not‐lung, including regions of organ overlap, was performed as a binary classification task. A separate image‐segmentation classifier was trained on balanced labels for each pressure (6, 8, 10, and 12 cmH_2_O) and for each mouse (*N* = 8) as the single mouse held out for cross‐validation. Thus, a total of 32 distinct classification experiments were performed. Each such training procedure completed in approximately 30 sec on a desktop computer. For each pressure, the cross‐validation accuracy was found to be 0.888 ± 0.0191, 0.909 ± 0.0144, 0.919 ± 0.0120, and 0.922 ± 0.0107, respectively. This result indicates that misclassifications between the interior texture (lung) and exterior texture (not‐lung) are less likely to occur at higher pressures. The corollary is that the difference between lung and not‐lung image texture is greater for higher pressure than for lower pressure. In other words, lung texture contrast increases with pressure. Regardless of pressure, the overall accuracy is excellent with only about 11% of the entire image being misclassified in the worst case. Additionally, the consistently low standard errors indicate that relatively few animals are required in order to train the segmentation classifier.

**Figure 4:**
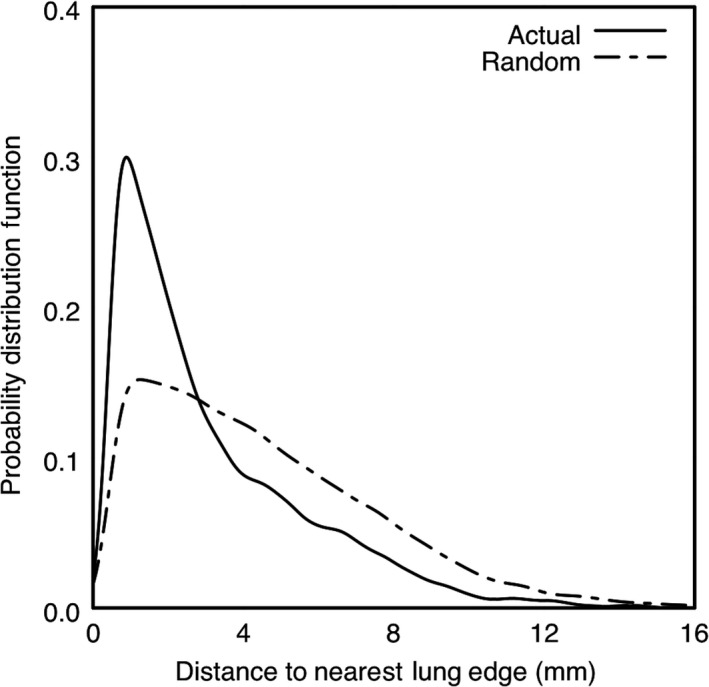
Nonrandom distribution of misclassifications. The smoothed kernel density distribution of distances between lung boundary and misclassifications is shown (solid). The distribution is skewed such that the majority of misclassifications occur within a few millimeters of lung boundaries, where image texture often is visually ambiguous as well. For reference, the distribution of distances for the scenario of randomized misclassifications is given (dashed)

It was observed that the locations of misclassifications within the images were not random. A smoothed kernel density distribution of the distance between the center of a misclassified image window and the center of the nearest window known to be lung is shown as the solid curve in Figure 4. There, it is seen that, across all mice, the overwhelming majority of misclassifications occurred very near a lung boundary. This indicates that the bulk of the lungs can be identified accurately by the proposed method.

**Figure 5:**
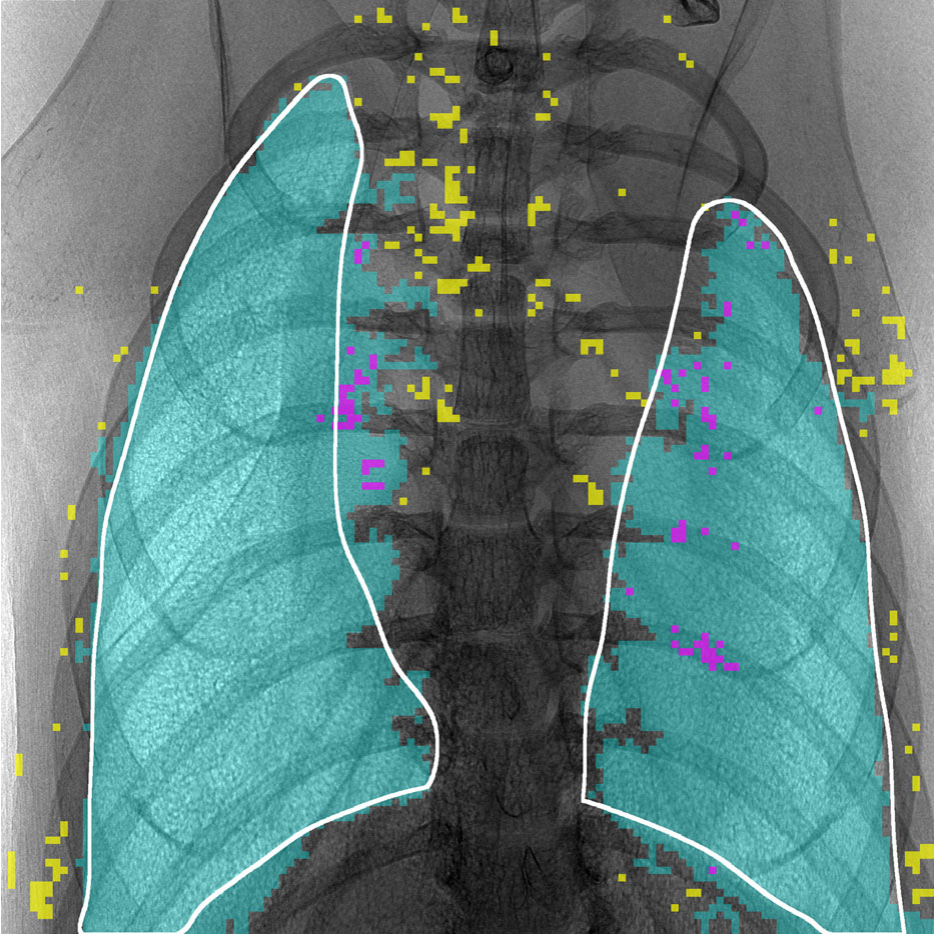
Automatic segmentation of a hold‐out image. The white curves indicate one possible manual delineation of regions with distinct lung texture. The cyan overlay indicates overlapping convolution windows predicted by the trained classifier to be lung. The magenta and yellow overlays indicate, respectively, regions included (as being lung) or excluded (as being not‐lung) during optional post‐processing. Most of the windows overlaying the heart are selected as are only very few windows overlaying the rib cage. Thus, this segmentation method is expected to have both high precision and high recall

To illustrate this more concretely, the result of applying a trained image‐segmentation classifier to an entire hold‐out image is shown in Figure 5. That is, each 32x32‐pixel window of this new image was “shown” to the classifier trained on other images which then returns a probability that the window corresponds to lung (as that label was defined for training). In Figure 5, the cyan color indicates windows declared to be lung and the white curve indicates a lung boundary visually inferred from the original image. Note that there are relatively few obvious misclassifications and that those regions tend to be small and isolated. To better illustrate this, we applied a region‐size exclusion filter to the declared lung windows. This may be done in Fiji by first applying the “Fill Holes” algorithm from the Binary submenu and then using “Analyze Particles” to create an exclusion mask of all remaining disconnected regions. Those windows included via post‐processing as being lung are indicated in magenta in Figure 5 and those windows excluded as being not‐lung are indicated in yellow. For the image shown, post‐processing increased the overall segmentation accuracy from 93.4% to 96.1%. It is here emphasized that this post‐processing is optional and task‐specific; excepting the preceding sentence, the classification accuracies given throughout the text are for images *without* post‐processing. It is again seen that the persistent misclassifications tend to occur near the lung boundaries where there may genuinely be lung (in projection) but can be difficult even for a human observer to visually identify as such. Still, the cyan regions overwhelmingly colocate only with the regions expected to be lung according to both anatomy and previously published XPCE imaging theory.

### Regional variations

**Figure 6:**
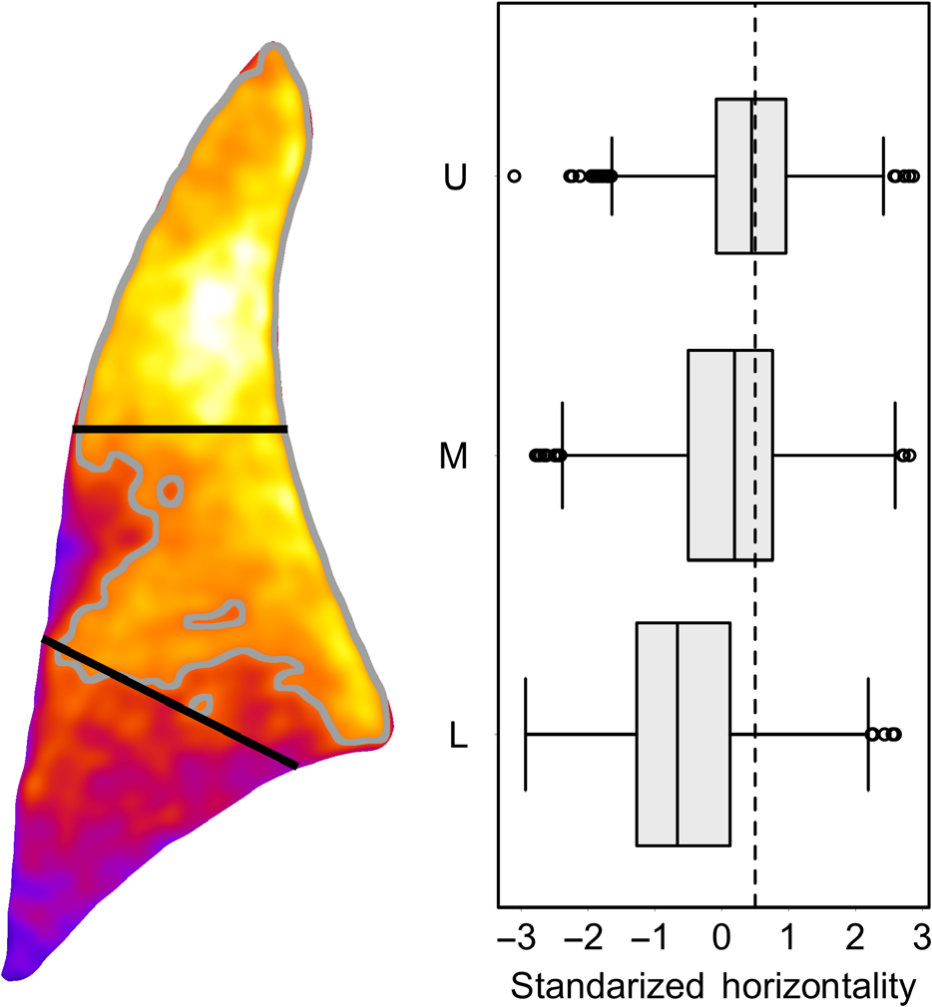
Unsupervised clustering within the right lung. For illustration purposes, a heat map (left) of the standardized horizontality for each right lung region image at 10 cmH_2_O was mapped to a common atlas and averaged. This was done to visualize, at once, lungs of different size and shape. Upper (U), middle (M), and lower (L) regions are defined here as the distinct k‐means clusters and do not perfectly colocate with distinct lobes. A box plot (right) of the horizontality in each region, prior to any image deformations, for all mice imaged at 10 cmH_2_O. The dashed line indicates the mean value of the vertical–horizontal threshold after standardization to z‐scores

For each mouse, the texture orientation and horizontality were computed for each square window as described in the Methods. A spatial map of the standardized horizontality was created for each right lung. For illustration purposes only, each such map was registered to the same atlas lung and averaged together. This was done for the right lung only in an effort to minimize the potentially confounding effects of heart motion on the regional texture differences within the left lung. The result is shown at the left of Figure 6 where it is seen that horizontality values above the median value (indicated by the gray curve) overwhelmingly cluster in the upper and middle portions of the lung. This unsupervised clustering of horizontality values is illustrative of the image texture difference along the cranial–caudal axis. The black lines indicate regions defined by an independent k‐means clustering of the window locations where the horizontality was computed. The lines do not indicate the boundary of any particular lobe but do indicate roughly where one should expect to see differences in texture that potentially correspond to the accrued projected structure of the distinct lobes. Indeed, a box plot of the standardized horizontality values computed individually for all mice imaged—prior to any image deformation or averaging of co‐registered images—indicates that the horizontality is distinct in the upper, middle, and lower regions of the right lung (Fig. 6, right). Additionally, the rank correlation (assessed via Kendall’s Tau within the right lung at 10 cmH_2_O) of horizontality with the median window intensity is only τ = −0.124. Similar box plots and low magnitude rank correlations were found for each imaging pressure (data not shown). Taken together, these results provide evidence that the cranial–caudal variation in quantified image texture is a genuine feature of the projection image that is distinct from the regional attenuation variances one might expect due to size or shape of the lung.

### Relation to pressure

**Figure 7:**
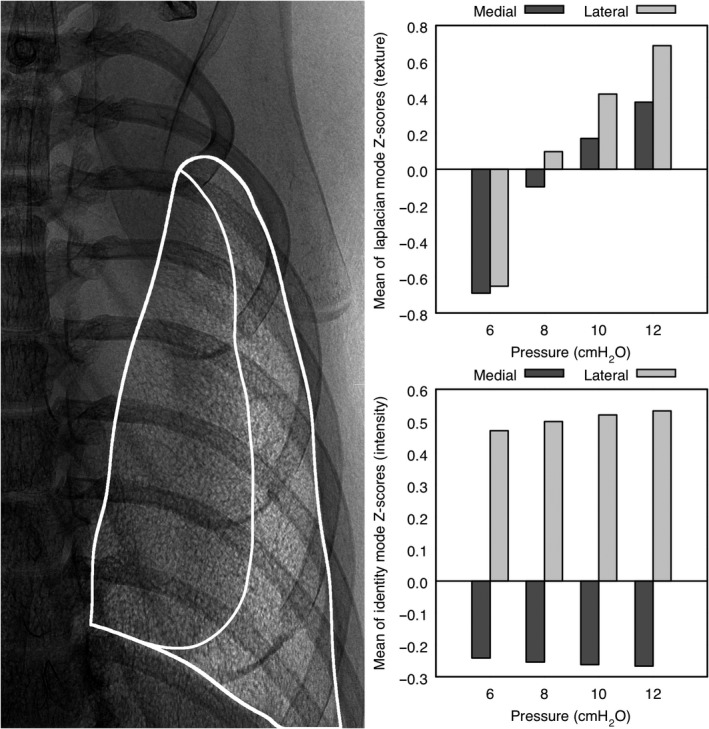
A comparison of between intensity contrast and texture contrast for the same mouse shown previously. At the left is shown the outer boundaries of the left lung and the inner boundary defining where lung overlaps the average position of the beating heart. In the top bar plot, the texture statistic is seen to vary monotonically, and to much greater magnitude, for both medial and lateral regions than does the grayscale image intensity

Because increased inspiratory pressure mechanically increases lung volume, we imaged mice at purposely chosen inspiratory pressures. This was a means of repeatably defining known states of lung expansion and, thus, well‐defined states of image texture. Here, it is not necessary to know a precise mapping of pressure state onto texture state but only that the texture states likely are distinct. Each mouse was imaged in an upright position during a 4‐sec breath hold. The ventilator pressure during each breath hold was selected at random from 6, 8, 10, or 12 cmH_2_O. It was found that the median value of the Laplacian mode window—which here describes the total intensity of chiefly edges within the window—is proportional, on average, to the ventilator pressure. This result is shown as the bar plot at the top, right of Figure 7. For comparison, a measure of the grayscale intensity over the same regions is given at the bottom, right. To appreciate the differences, consider the boundaries drawn on the XPCE image of the left lung given at the left of Figure 7. The darkened region on the medial side of the lung is the portion of the lung that overlaps the beating heart in projection. It is seen in the bar plots that the change in the mean texture statistic with maximal change in pressure is about 200% whereas the analogous change in grayscale image intensity is less than 20%. Furthermore, the large, monotonic change in this particular texture statistic persists similarly in both medial and lateral regions. This is an important observation indicating that lung structure expected to change with pressure can be quantified via image texture even through substantial overlap (in projection) with other organs.

The capacity of image texture to indicate distinct expansion states was investigated first as a binary classification task within only the right lung such that potential differences between lobes could be tested. It should be emphasized that, in general, there is no single scalar that adequately describes an image texture, and therefore ensemble learning comprising many predictors that capture various aspects of image texture to varying degrees was employed. As described in the Methods, a classifier distinct from previous ones was trained to predict inflation pressure—that is, a tunable surrogate measure of the expansion state of lung microstructure—from the image texture statistics. The binary classification results are shown in Table 1. For all such comparisons, the accuracy is greater than 50% which indicates that the *collective* verdict of all image windows queried is the correct pressure. Note that the prediction accuracy increases with the size of the pressure difference. This suggests that lung image textures are less distinguishable at lower pressure differences. A consistent finding is that the accuracy in the lower region was slightly less than that of the upper region. Taken together, the results indicate that even a well‐validated classifier trained specifically for one region or for one pressure difference may not be as effective when applied to another region or pressure difference.

**Table 1:** Accuracy of paired pressure classifications within the right lung, by region

	Accuracy					
Pressure (cmH_2_O)	6‐8	8‐10	10‐12	6‐10	8‐12	6‐12
Upper	0.690	0.658	0.660	0.835	0.787	0.884
Lower	0.662	0.650	0.620	0.804	0.757	0.874

The relation between image texture difference and pressure difference was explored another way. A single classifier was trained to identify windows within both lung regions, including regions of possible overlap with organs in projection, as corresponding to one of the four known pressures. That is, the image texture statistics were the predictors and the outcome was any one of the four discrete pressure levels. Similar to previous experiments, this was repeated once with each of the mice held out, in turn. The ensemble‐averaged confusion probabilities for these experiments are given in Table 2 for each lung. The rows correspond to known pressures and the columns correspond to the average probability of the classifier choosing a given pressure. For example, for the right lung, the entry for row 6 cmH_2_O, column 10 cmH_2_O indicates there is about 10% chance that the classifier will declare a window to be pressure 10 cmH_2_O when it actually is pressure 6 cmH_2_O. Whenever the diagonal element is the greatest element per row, the collective verdict of all image windows queried is correct. This is seen to be the case for all pressures and in both lungs, regardless of overlap and therefore the classifier can be used to choose the correct inflation pressure of the entire lung from feasible alternatives. The previous trend in decreased misclassification with increased pressure difference is again seen. For example, for the right lung, when an image window actually corresponds to pressure 10 cmH_2_O, there is a substantial chance (23%) of confusing it for a typical 12 cmH_2_O window, but only about 4% chance of confusing it for a 6 cmH_2_O window. This result is consistent with visual inspection of the image texture changes with pressure (see online Supplement https://doi.org/10.7910/DVN/WULRFA) (Brooks, 2019).

**Table 2:** Ensemble‐averaged confusion probabilities for multi‐class pressure classifications within each entire lung including projection‐overlap regions which can be substantial for the left lung. For each true pressure row, the probability of selecting a pressure is given in each column

	Right lung				Left lung			
cmH_2_O	6	8	10	12	6	8	10	12
6	0.635	0.238	0.104	0.0230	0.645	0.245	0.0857	0.0242
8	0.271	0.354	0.294	0.0811	0.252	0.406	0.268	0.0746
10	0.0418	0.214	0.515	0.229	0.0390	0.206	0.538	0.217
12	0.0208	0.0979	0.286	0.595	0.0248	0.100	0.277	0.598

## Discussion

### Relation of image texture to lung structure and function

There are numerous examples in the literature of the analysis of image texture as it can be seen in radiographs and in various CT slices. The images we analyzed differ because of the twofold effect of an extended object‐to‐detector free‐space propagation distance in the so‐called near‐field regime. For our detector size, source placement and source power, it is possible to create an imaging geometry with a magnification factor of four on a single laboratory table; this alone is a substantial improvement over single tomographic views from conventional micro‐CT systems that typically have a magnification of approximately one. Additionally, the extended propagation distance enables detection of the characteristic edge‐enhancement associated with XPCE imaging. Together, the improved resolution and enhanced edges reveal structure within the lung that appears in projection as the image texture we analyzed.

It is thus expected that as the structure of the lung changes, so does the observed image texture. Indeed, textural changes were noted with lung inflation and with increasing inspiratory pressure, presumably due to expanding lung airways. Thus, for our purposes, one way to impose a controlled change in structure is to inflate the lungs to various known pressures. Analyzing the appearance of defined structural changes may be an avenue for assaying regional changes in physiological functions. For example, because the right mouse lung comprises four distinct lobes that can differ in compliance, ventilation, and blood flow (Irvin and Bates, 2003; Sato et al., 2015; Meyerholz et al., 2018), the capacity of the microstructure to expand in three‐dimensions varies from lobe to lobe. One might therefore expect some corresponding difference in the image texture observed in a projection image. Indeed, we observed a regional variation in image texture consistent with the known structure of the right lung. In Figure 6, the box plot of the horizontality indicates that the upper portion of the right lung – which (in projection) corresponds most with the cranial lobe – does not have a single, strong orientation, and thus is more randomly oriented. In contrast, the texture orientation of the lower region is definitively more vertical than horizontal. Furthermore, the middle region appears as a transitional region. This is consistent with the fact that that region comprises projections through multiple lobes (cranial, middle, and caudal). Thus, we observe a change in image texture for expected overlays of the lung structure which includes large vessels and large airways in the central region and proximal region and small airways, capillaries, and alveoli in the distal lung. We emphasize that this one image texture statistic should not be thought of as a direct quantification of any physiological function or physical property. Instead, we claim only that the observed cranial–caudal trend is consistent with known geometric differences within the upright lung.

We imaged mice in the upright position during an inspiratory hold at various pressures. Our assumption was that the response of the live mouse would be repeatably, distinctly different at 6 cmH_2_O inflation that at 12 cmH_2_O inflation, for example. Indeed, we found that a random forest classifier is able to reliably distinguish these pressures solely from rudimentary image texture statistics. We hypothesize that the textural changes are the result of regional changes in volume, for example, as occur with the expansion or opening of acinar units and alveoli. We stress that all such pressure classifications are done via plurality‐vote classifiers as applied to the entire predefined lung regions. This means that a non‐negligible percentage of the individual 32x32‐pixel image windows are misclassified but that the largest predicted class is indeed the correct pressure class. We found that these results hold for a wide variety of pressure classifications and for all mice tested.

### Image texture changes

The preceding arguments are not, and are not intended to be, any quantitative definition of a specific texture–structure relationship or any assessment of the relative contributions of macroscopic versus microscopic structures to the texture observed. Instead, the choice of inflation pressures was an empirical one where we visually observed texture differences in preliminary images of separate animals. We thus used the pressure as a means of defining repeatable “texture states” which plausibly relate to a range of physiological conditions. Given that we were able to accurately classify pressure from only the appearance of texture, a pertinent question then is: by how much does the image texture change for a given change in lung structure (or pressure, for example). This question is challenging for several reasons. In general, there is no single scalar value that adequately conveys the notion of image texture. Additionally, even when a particular numerical definition of texture is applicable, it is often the case that commonly employed quantification statistics depend nonlinearly upon one another. The consequence of this is considerable difficulty in assessing the true predictive capacity of arbitrary selections of texture statistics in regression models applied to heterogenous populations. Furthermore, as image textures, and texture statistics become more sophisticated, the number of realizations required to analyze them meaningfully increases as well. Therefore, even in cases where a well‐defined preclinical outcome can be controlled, developing a regression map between quantified texture, and specified outcome can be challenging and both predictiveness and repeatability can be low. For these reasons, we chose to frame our analyses as categorical classifications rather than as regression models.

### Classification accuracy

To segment the entire XPCE images at once, we, in effect, used several images to train a classifier to recognize the low‐level statistical signature of a given image texture and then scanned a new image for that signature. The capacity for the user to purposely define textures of interest in some mice and then objectively identify similar regions in new mice is a key strength of the chosen segmentation method. For example, one might define only right lungs as the positive training label and leave left lungs unlabeled in order to explore the textural differences between the lungs within a single animal. The example shown in Figures 5 is just one of many reasonable classification tasks that could be defined for our image data.

It is important to recognize that the ability of human observers to visually assign legitimately correct labels varies with the task at hand and incorrect training labels will reduce the discriminatory power of the classifier. For example, if the white curves in Figure 5 define the correct lung boundaries, then some false negatives (i.e., missed lung texture) appear near the left lung apex and again at the rib‐spine interface near the middle portion of the left lung. That is, the expected labels were not predicted accurately in these regions. However, the given labels themselves may be incorrect. Consider that the visually identified, left lung boundary (white curve) was inferred by a human extrapolating from the apex and exploiting anatomical knowledge of where the lung boundary might be in projection. It is possible that the extremely faint texture near the left apex genuinely may not correspond to any appreciable anterior–posterior depth of lung. In other words, in this case, it is possible that the classifier predictions are correct where the training labels are not. Another example of this might be where some regions of the right lung overlying the diaphragm have been predicted to be lung. With respect to the given labels, this prediction is a false positive; however, with respect to anatomy, this is not necessarily incorrect; in projection, there really can be a substantial overlap with the diaphragm. In other words, the diaphragm obscures the posterior regions of the inferior lung and the extent to which lung texture must be seen in order to statistically count as lung is not known.

Once the training labels are stipulated, the choice of image statistics becomes important to segmentation accuracy. There is a vast literature on image texture analysis, computer vision and the extraction of quantifiable features from grayscale images. Therefore, there likely are other statistics well‐suited to the analysis of lung image texture for specific purposes. We chose the statistics that we did precisely because they are simple and readily calculable. We found (data not shown) that many, much more sophisticated statistics, such as those based on the so‐called structure tensor ( Jähne, 2004) or derived from gray‐level co‐occurrences matrices ( Haralick et al., 1973), were strongly correlated with the rudimentary ones that we ultimately employed. In contrast to their more sophisticated cousins, the texture statistics chosen here are: easy to compute in a wide variety of software packages, straightforward to interpret in terms of the fundamental properties of all image textures (Ravishankar Rao, 1990; Jähne, 2004) and generally amenable to the calculation of confidence intervals.

That the image texture can be found and analyzed though the ribs (in projection) is another key strength of the ensemble learning segmentation method applied to lung images. Even while controlling for differences in grayscale intensity distribution, the obviously darker, and strongly spatially correlated, rib regions effectively always represent edges and thus, in comparison to the rest of the image, one expects substantial differences in the spatial derivative at and/or within the rib regions. Because the gradient is a fundamental property of any image texture, many texture statistics are strongly sensitive to the ribs and therefore two‐dimensional plots of said texture statistics can convey a strong visual impression of ribs. In other words, heatmap‐style plots of some individual texture statistics are inconsistent with the observation that the image texture corresponding to lung passes unimpeded through the ribs. The well‐established ensemble learning method we employ comprises several weak learners – each of which might be “fooled” at the ribs but perhaps in subtly different ways – into a single classifier that correctly identifies both rib and non‐rib regions within the lung as having the same texture.

### Model and predictor reproducibility and applicability

For all classification models, we employed a cross‐validation scheme that left one mouse out of training – as if we had only *N‐*1 mice – and then used the *N*th mouse as the validation data upon which the trained classifier was tested. This was repeated for all mice, resulting in an eightfold cross‐validation. In this scheme, the numerical decision boundaries for one set of mice are to be applied to a separate mouse. Therefore, we converted all image‐derived statistics to a standardized z‐score on a per mouse basis. This way, each statistic can be compared directly, despite any potential bias in values for individual mice (as might occur if, for example, a mouse had uncommon anatomical measurements).

In the case of the segmentation classifier, the mean accuracy was very high and the standard error relatively low, for all pressures tested. This indicates an interchangeability of the mice in the training data, which bodes well for the applicability of similar classifiers to new mice. Incidentally, we also note that for the specific purpose of image segmentation, the performance of a classifier trained at given pressure was nearly matched by classifiers trained at other pressures; this suggests that once the intra/extra‐lung texture difference becomes detectable, it remains so for all pressures tested.

In the case of the intra‐lung pressure classifier, regional differences, the inflation pressure and inflation pressure difference each are important. Our results show that different inflation pressures can be identified from observable image texture. As the pressure difference is increased, the distinguishability of the textures increases. Additionally, a pressure difference of 2 cmH_2_O is less reliably distinguished at lower pressures than at higher pressures. Together, these observations suggest that the image texture within the lung regions is not as well‐defined at low pressures than it is at higher pressures and is consistent with both visual inspection of the images (see online Supplement https://doi.org/10.7910/DVN/WULRFA) (Brooks, 2019) as well as the association of image texture with lung microstructure which necessarily is less expanded at lower pressures. Although the binary pressure classification accuracy was seen to differ regionally (Table 1), this in no way implies that the pressure differs regionally or that we have developed a regional measure of pressure. Instead, the *classifier* differs regionally. That is, the numerical decision boundaries applicable to the upper region may not always be applicable to the lower region. Thus, the classifier we describe likely would have to be retrained in order to assess mice imaged under more natural breathing conditions. A rigorous assessment of how many mice would be required for training, for example, a classifier capable of distinguishing various respiratory phases during free‐breathing is beyond the scope of the present work but is a logical next test of the method presented.

### Potential for physiological assay

The results given in Table 2 are encouraging for the broader application of the image texture analysis of XPCE projection images to lung physiology. Using only basic texture statistics as predictors, all pressures were correctly classified from a natural range of choices, for both lungs, regardless of overlap with nonpulmonary structures – this is key. One can therefore envision locally altering the lung via a disease model such as induced chronic obstructive pulmonary disease (Wright et al., 2008) or induced pulmonary fibrosis (Moore et al., 2013). One then could train a classifier on manually defined small regions of suspected lung damage and then effectively scan new animals for that damage via statistical similarity in training texture. Because states of “normal” image texture within the whole lungs was so reliably classified in the projection images, we speculate that only relatively few, relatively small regions of interest will be required to accurately identify diseased regions in new animals. Thus, the image texture analysis of high‐magnification XPCE projection images potentially opens a new avenue for studying the onset and treatment of lung disease in preclinical models.

### Phase‐contrast enhancement

The X‐ray phase‐contrast enhanced (XPCE) projection images we analyzed are mixed‐mode images in the sense that they comprise a dominant attenuation component and an unquantified phase component. The edge‐enhancement generally associated with propagation‐based X‐ray phase‐contrast imaging occurs at interfaces between media of differing refractive decrement. In the lung, such an interface is the boundary between the air‐filled alveoli and the surrounding capillaries and parenchymal tissue. As the lung comprises a vast number of such interfaces, one might expect numerous enhanced edges to be present at the plane of the distant detector. However, because the interfaces themselves are distributed (pseudo‐)randomly throughout the lung, it is unclear to what extent correlated phase components contribute to the two‐dimensional intensity distribution ultimately detected.

As described in the literature, it is possible to separate the attenuation and phase components of image intensity for a variety of imaging scenarios. For our free‐space propagation scenario, some sort of phase‐retrieval algorithm is required to explicitly quantify only the phase component (Gureyev et al., 2009; Pelliccia et al., 2018). While a comparison of competing phase‐retrieval methods is well‐beyond the scope of this work ( Burvall et al., 2011), the upshot is that so‐called single‐shot phase‐retrieval methods require some a priori estimation of the spatial distribution of the complex refractive index throughout the object imaged. This is challenging for an object as intricate as a real lung and further complicated by the dependence of the complex refractive index upon the wavelength of the X‐ray beam, which is not a single value for our polychromatic laboratory source. It is important to note that we directly analyze the same phase‐contrast‐enhanced image data that would be input into a phase‐retrieval algorithm but without imposing a prior distribution upon the complex refractive index or, stronger still, without making any presumptions at all about the relative contributions of phase or attenuation to the observed image texture. For this reason, our texture analysis is simpler, more general, and thus likely is more reproducible across animals than if we had applied a particular phase‐retrieval method to the image data.

## Conclusion

x‐ray phase‐contrast enhanced (XPCE) projection images of in vivo mouse lungs were acquired using bench‐top imaging system and laboratory X‐ray source. A rudimentary analysis of the distinctive image texture manifest in the XPCE projection images was performed. The image texture statistics employed are simple and reproducible across mice. Various image classification tasks were designed using these statistics as predictors. The results show that lung image texture can indicate mechanical ventilation pressure, which was employed as a repeatable surrogate measure of the expansion state of the microstructure. Additionally, quantifiable differences in lung image texture were observed to vary along the cranial–caudal axis in upright mice, as is expected from known anatomy. Importantly, the image texture‐based classifications were consistent even in areas of highly attenuating structures such as ribs, the mediastinum and the heart. Therefore, the analysis of lung image texture as seen in high‐magnification XPCE projection images represents a promising avenue for the low‐dose, noninvasive study of regional, in vivo lung function in preclinical animal models.

## Conflict of Interest

The authors declare no conflicts of interest with publication of this manuscript.
